# Extended Spectrum β-Lactamase–producing *Escherichia coli* in Neonatal Care Unit

**DOI:** 10.3201/eid1611.100366

**Published:** 2010-11

**Authors:** Sarah Tschudin-Sutter, Reno Frei, Manuel Battegay, Irene Hoesli, Andreas F. Widmer

**Affiliations:** Author affiliation: University Hospital, Basel, Switzerland

**Keywords:** Outbreak, transmission, extended-spectrum β-lactamase, ESBL, Escherichia coli, neonatal care unit, nosocomial infections, bacteria, Switzerland, dispatch

## Abstract

An outbreak of extended-spectrum β-lactamase–producing *Escherichia coli* in a neonatal care unit began with transmission from a mother to her newborn twins during vaginal delivery. Subsequently, infection spread by healthcare worker contact with other neonates; a healthcare worker also was infected. Knowledge about transmission may improve infection control measures.

Gram-negative *Enterobacteriaceae* expressing extended-spectrum β-lactamase (ESBL) are among the most multidrug-resistant pathogens in hospitals and are spreading worldwide (*1**–**3*). Infections caused by ESBL–producing organisms have resulted in poor outcomes, reduced rates of clinical and microbiological responses, longer hospital stays, and greater hospital expenses (*4**,**5*). Multiple outbreaks of ESBL-producing *Enterobacteriaceae* in intensive care units (ICUs) and increased rates of illness and death, especially in neonatal ICUs, have been reported (*6**–**10*). Physical contact is the most likely mode of transmission. The gastrointestinal tract of colonized or infected patients is the most frequent reservoir. Several studies indicate that transient carriage of bacteria on the hands of healthcare workers (HCWs) may lead to transmission to patients (*7**,**11*).

We report an outbreak of ESBL-producing *Escherichia coli* (ESBL *E. coli*) in a neonatal intermediate care unit. Initial transmission was from a mother to her newborn twins and subsequently by physical contact of HCWs with other patients; an HCW also was infected.

## The Study

The Department of Obstetrics and Gynecology of the University Hospital, Basel, Switzerland, has 94 beds; ≈2,000 babies are delivered there each year. The neonatal unit includes 12 beds for healthy newborns and 9 beds for infants requiring intermediate care.

A 29-year-old woman with dichorionic twin pregnancy was admitted to the antenatal care unit at 32 weeks’ gestation because of spontaneous preterm rupture of membranes of the first twin. Her medical history was unremarkable. Screening results for gestational diabetes, as well as urinary controls and vaginal swabs for group B *Streptococcus*, were negative. After confirmation of preterm rupture of membranes by ultrasound and vaginal examination, therapy was initiated with amoxicillin/clavulanic acid (3 × 2.2 g/d) for 10 days, tocolysis with betamimetics (hexoprenaline) until 34 weeks’ gestation, and 1 course of steroids for lung maturation (betamethasone 2 × 12 mg with an interval of 24 h).

Five weeks later, the woman spontaneously delivered 2 healthy boys (1,920 g, Apgar scores 9/10/10; and 2,045 g, Apgar scores 8/9/9) under epidural analgesia with placement of a urinary catheter. Two days after delivery, an asymptomatic urinary tract infection with ESBL-*E. coli* was detected in the mother; it was treated with trimethoprim/sulfamethoxazole for 7 days. Follow-up urinalysis was negative for ESBL *E. coli*; however, rectal swab performed to document colonization was positive for ESBL *E. coli*. This pathogen persisted for >7 months after delivery, after which the patient was lost to follow-up.

Both twins were initially admitted to the neonatal intermediate care unit because of their prematurity. Six days after birth, screening rectal swabs confirmed colonization with ESBL *E. coli* in both neonates. The twins did not show clinical signs of infection and were discharged on their 20th day.

Screening of the 6 other neonates in the neonatal intermediate care unit during the twins’ stay showed that 3 were colonized. In addition, rectal screening of 31 HCWs indicated that 2 (7%) were positive for ESBL *E. coli*. Invasive infection did not develop in any of the 3 neonates colonized with ESBL *E. coli*.

Monthly follow-up screening was performed for the 2 HCWs who were positive for ESBL *E. coli*. They continued working after reeducation about general hygiene precautions. One HCW left her job at the hospital and was lost to follow-up; the other was negative for ESBL *E. coli* at 2-month follow-up.

Rectal swab specimens for surveillance of intestinal carriage were obtained from all patients in the neonatal intermediate care unit during the outbreak and at 2 weeks, 5 months, and 7 months after the outbreak. Screening for ESBL *E. coli* carriage among HCWs was performed by obtaining rectal swabs.

Cultures were performed by using CHROMagar orientation medium (Becton Dickinson BBL Diagnostics, Sparks, MD, USA). ESBL production was identified according to the guidelines of the Clinical Laboratory Standards Institute (*12*). Routine susceptibility testing was performed by microbroth dilution (Micronaut-S; Merlin, Bornheim-Hersel, Germany). Four cephalosporins (cefpodoxime, ceftriaxone, ceftazidime, and aztreonam) were used for screening. If >1 of the cephalosporins showed increased MICs, ESBL *E. coli* was confirmed with Etest strips (AB Biodisk, Solna, Sweden) containing cefotaxime or ceftazidime, each with and without clavulanic acid.

Molecular typing was performed by pulsed-field gel electrophoresis (PFGE). ESBL was molecularly confirmed by PCR amplifying genes for TEM, SHV, and CTX-M β-lactamases. Amplicons were sequenced by using an ABI 3130 Genetic Analyzer (Applied Biosystems, Foster City, CA, USA).

**Figure 1:**
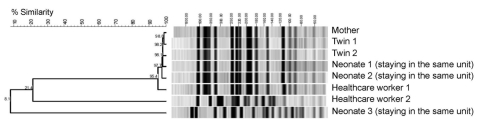
Molecular typing of extended-spectrum β-lactamase–producing *Escherichia coli* isolates by pulsed-field gel electrophoresis. Dendrogram shows a cluster of 6 isolates with identical banding pattern and 2 isolates with 2 distinct patterns.

**Figure 2:**
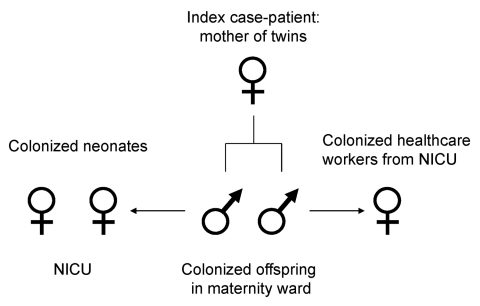
Spread of extended-spectrum β-lactamase–producing *Escherichia coli* outbreak. NICU, neonatal intensive care unit.

Genotyping by PFGE showed 1 dominant ESBL *E. coli* strain; 2 different genotypes were found in 1 HCW and in 1 of the screened neonates staying in the same unit as the twins (Figure 1). The outbreak strain was found in the index patient, her twins, 2 neonates staying in the neonatal intermediate care unit at the same time, and 1 HCW (Figure 2). Sequencing of the ESBL gene showed TEM-29 type. Surveillance cultures performed on all patients in the neonatal intermediate care unit indicated no further ESBL *E. coli* was present at 2 weeks, 5 months, and 7 months after the outbreak.

Before the outbreak, a quaternary ammonium–based disinfectant was used daily to clean the neonatal unit. HCWs routinely cared for healthy babies without using gloves but did use an alcohol-based hand sanitizer. Products for patient care were shared among neonates; in particular, no protective covering was used for clinical thermometers.

After screening showed ESBL *E. coli*, reinforced infection control strategies were established. A schedule of training sessions emphasizing proper hand hygiene, routine use of protective covering for clinical thermometers, environmental cleaning using an aldehyde-based disinfectant, and routine use of gloves and gowns for any patient contact (particularly changing diapers) was instituted. Furthermore, separate care products were used for each neonate.

## Conclusions

We report an outbreak caused by transmission of ESBL *E. coli* from a mother to her newborn twins and subsequent spread to other neonates and 1 HCW. The mother was most likely colonized before hospitalization, and a urinary tract infection developed peripartum. Transmission by contact during vaginal delivery of the twins and transmission by physical contact to 1 of the HCWs and the other neonates was the most likely mode of transmission. We interpret the detection of ESBL *E. coli* infection in 1 of the neonates and the other HCW as a coincidence because both had a different genotype (TEM-12) and PFGE pattern type of ESBL *E. coli*.

Because we screened only for ESBL *E. coli*, we might have underestimated the true extent of the outbreak. However, the ESBL-encoding gene, which is on a plasmid, could have been transferred to other *Enterobacteraceae* and would have been missed. Risk factors for colonization in newborns include low birthweight, duration of hospitalization, total parenteral nutrition, previous use of antimicrobial drugs, and mechanical ventilation in a neonatal ICU (*13*). In the intermediate care setting, breastfeeding was associated with a lower risk for ESBL-producing *Enterobacteriaceae* (*14*) because breastfed neonates have more contact with their mothers and therefore are possibly less frequently handled by HCWs. Our patients had only 1 identified risk factor: the twins from the colonized mother had low birthweight; the other neonates had no risk factors. Improved infection control strategies may be necessary to limit spread of ESBL *E. coli* in maternity wards because transmission to neonates during delivery is possible. A feasible approach could be to screen mothers whose neonates need to be transferred to ICUs; an outbreak in this setting would be particularly harmful.
